# Determination of hemispheric emotional valence in individual subjects: A new approach with research and therapeutic implications

**DOI:** 10.1186/1744-9081-3-13

**Published:** 2007-03-06

**Authors:** Fredric Schiffer, Martin H Teicher, Carl Anderson, Akemi Tomoda, Ann Polcari, Carryl P Navalta, Susan L Andersen

**Affiliations:** 1 Department of Psychiatry, Harvard Medical School, and the Developmental Biopsychiatry Research Program, McLean Hospital, 115 Mill Street Belmont, MA 02478 USA; 2 Child Developmental Sociology, Faculty of Medical and Pharmaceutical Sciences, Kumamoto University, Kumamoto, Japan

## Abstract

**Background:**

Much has been theorized about the emotional properties of the hemispheres. Our review of the dominant hypotheses put forth by Schore, Joseph, Davidson, and Harmon-Jones on hemispheric emotional valences (HEV) shows that none are supported by robust data. Instead, we propose that individual's hemispheres are organized to have differing HEVs that can be lateralized in either direction.

**Methods:**

Probe auditory evoked potentials (AEP) recorded during a neutral and an upsetting memory were used to assess HEV in 28 (20 F) right-handed subjects who were either victims of childhood maltreatment (N = 12) or healthy controls. In a sub-population, we determined HEV by emotional response to lateral visual field stimulation (LVFS), in which vision is limited to one, then the other hemifield. We compare a number of morphometric and functional brain measures between individuals who have right-negative versus left-negative HEV.

**Results:**

Using AEPs to determine HEV, we found 62% of controls and 67% of maltreated subjects had right negative HEV. There was a strong interaction between HEV-laterality and gender, which together accounted for 60% of individual variability in total grey matter volume (GMV). HEV-laterality was associated with differences in hippocampal volume, amygdala/hippocampal ratios, and measures of verbal, visual and global memory. HEV-laterality was associated also with different constellations of symptoms comparing maltreated subjects to controls. Emotional response to LVFS provided a convenient and complementary measure of HEV-laterality that correlated significantly with the HEVs determined by AEPs.

**Conclusion:**

Our findings suggest that HEV-laterality, like handedness or gender, is an important individual difference with significant implications for brain and behavioral research, and for guiding lateralized treatments such as rTMS.

## Background

Sperry's split-brain studies have created an abiding interest in hemispheric differences in cognition [1]. There is also a vast literature on hemispheric differences in affect. At present there are three predominant hypotheses regarding hemispheric emotional valence (HEV). The first and earliest [2-5] states that the right hemisphere (RH) has a superiority over the left in processing emotions, especially negative emotions. The second model suggests [6] that the left frontal cortex is associated with positive, approach emotions and the right with negative withdraw emotions. The third [7], argues that anger (a negative but often approach emotion) is associated with the left frontal cortex, and so hemispheric valence should be based on the person's emotional motivation.

In this paper we present an investigation of whether negative HEV, as a dispositional trait, is right lateralized in some subjects and left lateralized in others, with the opposite hemisphere providing a positive perspective, and whether such laterality of negative HEV is associated with substantial differences in brain anatomy, functional activity, psychiatric symptomatology and therapeutic response.

### A review and criticism of the right hemisphere negative emotion model

In support of the RH negative emotions model, we published a report [8] in which we found that subjects with a history of trauma and psychotherapy but without current posttraumatic stress disorder (PTSD) had significantly more right hemispheric activity during a brief psychiatric interview, while recalling an emotionally upsetting memory, but not during the recall of a neutral memory. A control group had smaller changes in the same direction that were not significant. We used probe auditory evoked potentials (AEP) recorded over each hemisphere, as an index of hemispheric activation, during the two memory conditions. Prior studies suggest that probe AEPs recorded over both hemispheres at C3 and C4 give an indication of relative hemispheric activation during a task [8,9]. In essence, attention to the task is thought to cause an attenuation of AEPs in response to 3 Hz bilateral auditory clicks, and a difference in hemispheric activation during the task appears to be reflected in a greater AEP attenuation on that side.

Although we originally found that the RH was more active, on average, during the recall of the unpleasant memory, we did mention then that 3 of the 10 trauma subjects showed more left-sided activation during the unpleasant condition. In retrospect, we believe that by emphasizing the averaged results, we may have missed an important sub-population of subjects who did not conform to the averages. The current study, attempts to replicate the findings of the original study, and challenges hypotheses presenting a "one size fits all" model of HEV. Further, we attempt to show the advantages and implications of characterizing each subject's HEV.

Shortly after the publication of our study, Rausch et al [10] published an fMRI study of patients with PTSD that supported our finding that the RH is more active during negative emotions. However, in a follow up study [11], bilateral activations were found during the negative condition, providing evidence that contradicts a right hemisphere-specific hypothesis.

During the Wada test paradigm marked changes in affect have frequently been observed with the anesthetization of one hemisphere or the other, and in many reports [12,13] displays of negative affect have been associated with an awake right hemisphere and an anesthetized left in support of the first hypothesis. However, Stabell et al [14] recently reported a compelling and comprehensive study of 270 patients on whom they performed the test. They, like Branch and Milner [15], found that individuals who manifested lateralized emotional responses during the Wada procedure had mostly positive emotions, which along with negative ones were distributed equally between the left and right hemispheres.

The right-hemisphere hypothesis received support also from Robinson's reports [16,17] that left-sided stroke was more often associated with subsequent clinical depression than right-sided strokes. However, a recent meta-analysis of 143 reports by Carson and associates [18] found no support for the idea that the risk of depression was related to the location of the lesion, including frontal lesions. In response to Carson, Robinson and his associates [19] reported their own meta-analysis, which found that there was a pooled correlation of r = -0.59 (R^2 ^= 0.35, p < .001) between the severity of depression and the proximity to the frontal pole of left lesions, but only a correlation of r = -0.17 (p = .17) for right-sided lesions. Implicit in Robinson's analysis is that although anterior left lesions may more often be associated with severe depression, many stoke survivors with right-sided lesions have depression. Robinson and associates' most recent clinical report [20] found that 9 of 12 (75%) patients with left, frontal stokes manifested depression while only 5 of 17 (29%) of patients with right-sided strokes did so. When they considered all left-sided strokes, only 10 of 22 (45%) had depression; they did not report data for right, frontal lesions.

### A review and criticism of the approach/avoidance hemispheric valence hypothesis

The second hypothesis, that the left frontal cortex is associated with positive, approach emotions and the right frontal, with negative, withdraw emotions has long been advocated by Davidson and his associates [6,21]. This hypothesis, which has evolved to incorporate more recent finding, is still based on earlier EEG studies on right-handed healthy females. In one study [22], an attempt was made to use a positive emotional film and a negative one to induce facial expressions reflecting happiness or disgust. The recorded facial expressions were then compared with lateralized frontal alpha EEG activity (LFA). Of 37 who entered the study, 11 were eliminated because of a lack of clear emotional facial expressions and 9 because of EEG artifact. During the happy facial expression condition, the *right *hemisphere showed somewhat more activity than the left. During the disgust facial expression, the RH was again more active, this time by a larger amount according to a graph of the mean log-transformed alpha power. Standard deviations were not reported. When LFA was compared between the whole positive and negative films, both of which elicited reports from the subjects of strong emotional responses in the expected happy versus disgust directions, no hemispheric EEG differences were found.

In a second study [23] baseline LFA among 43 healthy, right-handed women predicted negative affect to a film intended to be emotionally negative, but did not predict positive affect. The strength of the prediction was reported as a squared semipartial correlation of 0.14, p < 0.05, which indicates that it accounted for only 14% of the variability. Davidson and colleagues never discuss the possibility that their weak correlations may result from a subset of subjects with reversed laterality. In subsequent work Davidson and his associates have continued to indicate that an individual's LFA might predict his response to acoustic startle probes [24] and found no relationship between asymmetry at F3/F4 or F7/F8, but did find a relationship between asymmetry and log α^2 ^at FP2-FP1 with an R^2 ^of .17 (p = .02) for negative emotional images (after offset but not during picture presentation). Startle responses to positive pictures did not correlate with alpha asymmetry.

Davidson [6] found no difference in any gross morphometric measurement from MRI data between a group of extreme left- and right-frontally active subjects by LFA. Hagemann et al [25] attempted to replicate Davidson's reports of an association between LFA and emotional responses to affective slides. Using techniques that Davidson's group had used, they found no relationship when the 1990 procedure [23] was used, but found the opposite result (left LFA was associated with negative affect and right LFA was associated with positive affect) when a 1993 procedure [26] was employed. Hagemann applied 40 different analyses to the raw EEG data and did find that with a novel procedure using a C_Z _reference and 8 minutes of eyes-closed EEG recording, he replicated Davidson's hypothesis with an R^2 ^= 0.10. Hagemann cited six failed attempts to replicate this aspect of Davidson's work, and discussed serious methodological issues in using alpha EEG. Two PET imaging studies [27,28] did not support Davidson's hypothesis.

### A review and criticism of Harmon-Jones' variation of the approach/avoidance valence hypothesis

The third major hypothesis was put forward by Harmon-Jones [7] who proposed a variation of Davidson's approach/avoidance valence hypothesis. He hypothesized that negative emotions such as anger can be associated with approach, and that the motivational direction was more important than the positive/negative dimension. He suggested that many researchers associate the right frontal hemisphere with pathology and base treatments such as biofeedback on that assumption, but he cautioned that increased left hemispheric activity may not always be beneficial. Unfortunately, Harmon-Jones [29] reported only averaged data and did not include a measure of viability. His main result from 42 healthy female subjects showed that an anger-inducing condition was associated with greater left-frontal brain activation than a condition that did not induce anger, but his findings suggested that considerable inter-subject variability was present. In an earlier study [30] on 26 male and female adolescents, he found a significant correlation between anger scores and LFA with an R^2 ^= .24. While these findings are very important, they account for only 24% of the variability. Similarly, Hewig et al [31] reported a significant correlation between frontal asymmetry and anger-out with an R^2 ^value = 0.08.

### A review of meta-analyses of imaging studies related to hemispheric emotional valence

Murphy et al [32] performed a meta-analysis of 106 PET and fMRI studies of human emotions and found no evidence to support the hypothesis that the left and right brains were associated with positive and negative emotions respectively. The only positive finding was that "approach" emotions showed maxima in the left hemisphere more often than the right (L= 165, R = 134; p < . 05), but even with this finding 45% of the individuals showed greater right hemispheric activity during the "approach" emotions. These authors found no consistent left versus right differences in frontal cortical activation related to either approach versus withdraw emotions or between positive and negative emotions.

Phan and associates [33] found evidence for bilateral brain activations in a meta-analysis of 55 brain imaging studies during different positive and negative emotions. Eugene et al [34] reviewed the literature and presented their own data to argue that inconsistent results from fMRI studies of emotion (specifically sadness) seem to be related in large part to inter-subject variability that usually goes unreported. They suggest that individual data as well as group data be reported.

### A summary of our interpretation of the literature regarding the prevailing hypotheses concerning hemispheric emotional valence

We believe that none of the three major established concepts on HEV have strong or consistent empirical support and are contradicted by 1) meta-analyses of imaging studies [32,33]; 2) by a preponderance of Wada reports [13-15,35-37]; and 3) by reports that depression can be associated with strokes in either hemisphere [18,20]. Nonetheless, cerebral laterality remains important to psychology and psychopathology. Split-brain studies [1] have definitively demonstrated that each hemisphere is capable of simultaneous, autonomous mentation that can extend to the psychological properties of each hemisphere in a given split-brain patient [38,39]. Bogen [40,41] has suggested that in intact individuals, each hemisphere might be able to support an autonomous center for mentation associated with that hemisphere.

### Our alternative hypothesis for hemispheric emotional valence

From clinical observation consistent with these findings [42,43], we propose an alternative hypothesis for HEV that one hemisphere may have a more negative disposition and that the other may have one that is more positive, **but that the side with the more negative HEV varies among subjects**. ECT, transcranial magnetic stimulation (TMS) [44-46], deep brain stimulation, antidepressant drugs [47,48], benzodiazepines [49], and neuroleptics [50,51] may exert lateralizing effects, which could benefit some, but not others, due to these differences in hemispheric affective valence. HEV, if shown to be a valid concept supported by anatomical, functional and psychometric data, would likely be a valuable baseline variable in the evaluation of a wide range of research data and clinical practices, including psychotherapy [42] and psychopharmacology [47,48,50,52,53].

### Our present study to test our alternative hypothesis on hemispheric emotional valence: that negative emotion can be associated in a given individual, as a trait, with either the left or the right hemisphere

In addition to extensive psychometric evaluations, the subjects in the present study underwent brain anatomical MRI's from which hemispheric grey matter volume (GMV) was measured by two different programs, FreeSurfer (FS) and Voxel-Based Morphology (VBM), implemented in SPM2 [54]. For female subjects, we used the MRI's to evaluate the total volume (grey and white) of three regions of interest: the hippocampus, amygdala, and corpus callosum. Male and female subjects underwent also an echo planar imaging-based measurement of water proton transverse relation times (T2), which appear to estimate steady-state regional cerebral blood volume (rCBV) [55]. We conducted statistical analyses to determine if a history of abuse, gender, and/or HEV, as measured by AEPs, correlated with these any of these MRI-derived measurements.

On a randomly selected subgroup of our subjects, we compared the AEP results also with lateral visual field stimulation (LVFS), a simple technique to assess HEV that can be easily performed in the office [56]. LVFS is effected by the use of glasses that are taped to occlude either the left (LVF) or right visual field (RVF). LVFS has been demonstrated to induce significant alterations in affect in placebo-controlled studies [44,56,57]. According to studies using BOLD fMRI [58], theta EEG [57], and ear temperature changes [57], LVFS has induced significant increases in contralateral brain activity. LVFS has been found to predict the clinical response to a two-week course of left-sided rTMS in severely depressed patients [44]. Our hypothesis was that the data from the AEPs would correlate with those from LVFS, and that the agreement of two independent measures of HEV as well as correlations with our anatomical, functional, and psychometric measurements would lend support to our concept of individual hemispheric emotional valence and its relevance to research and practice.

## Methods

### Participants

Subjects were recruited via advertisements (i.e., bulletin board postings, newspaper ads, and subway & bus ads) for healthy right-handed individuals aged 18–22 years old, interested in participating in "psychiatric research". Seven hundred and thirty-two adults were initially screened with 1) telephone interviews to obtain basic demographic information and ascertain whether any exclusion criteria were met; 2) rating scales to assess current psychiatric symptomatology; and 3) questionnaires to ascertain family history of psychopathology and lifetime history of exposure to traumatic stressors. The primary entry criterion was a history of verbal or sexual abuse. Significant verbal abuse was defined as receiving a score of 40 or more on the Verbal Aggression Questionnaire [59], which assesses the frequency and degree of swearing, name-calling, criticizing, etc. that an individual receives from a family member. Sexual abuse was defined as 3 or more episodes of forced contact sexual abuse before their 18^th ^year and at least 2 years prior to enrollment. An abusive episode was defined as one in which the subject was "forced against her will into contact with the sexual part of her body or the perpetrator's body". The contact had to be accompanied by threats of harm to self or others, or feelings of fear or terror. Details of the both forms of abuse were ascertained through the use of the Traumatic Antecedents Questionnaire [60]. We have found [61] that verbal abuse has negative psychological consequences comparable to those associated with witnessing domestic violence or non-familial sexual abuse and larger than those associated with familial physical abuse, and so we combined our verbal abuse subjects with our sexual abuse group for statistical analysis.

Other criteria for participating included: 1) right-handedness; 2) absence of any alcohol, drug, or medication use for at least two weeks; 3) excellent hearing; and 4) good medical health. Potential subjects were excluded if they presented with a history of medical disorders (including neurological disease/insult, head injury, migraine headaches, and seizures); psychotic disorders; pregnancy; past or present alcohol/substance abuse; premature birth; complications during mother's pregnancy or delivery; in utero exposure to alcohol or drugs; a history of physical abuse (defined as any degree of intentional injury above the shoulders, or any intentional injury below the shoulders that received or should have received medical attention); or exposure to any other forms of trauma (e.g., motor vehicle accidents, natural disasters, near drowning, witnessing abuse, animal attacks, gang violence, etc.).

Of 64 right handed subjects recruited for imaging studies, 28 were studied for HEV using probe AEPs. This subgroup consisted of 16 (11F/5M) healthy controls, 9 (8F/1M) subjects with a history of sexual abuse and 3 (1FR/2M) subjects with high-level exposure to parental verbal abuse. Present or past history of DSM-IV Axis I Disorders were assessed using the Structural Clinical Interview for DSM-IV Axis I Disorders [62]. The subjects' social economic status (SES) was evaluated with Hollingshead's Index [63]. Fifteen of the 28 subjects (6 maltreatment with 4 females and 9 controls with 7 females) were tested also with LVFS as described in detail below. Table 1 shows the demographic information as well as administered tests for each participant. Subjects gave written informed consent and were paid for their participation. The study was approved and monitored by the McLean Hospital Institutional Review Board.

**Table 1:** Demographics and procedures for all study subjects.

Subj	Group	Sex	AGE	SES	Current Dx	AEP	Neuropsych Tests	GMV	T2	Hipp, Amyg	CC	LVFS
1	CSA	f	19	2	Maj Dep	Y	Y	Y	Y	Y	Y	Y
2	CSA	m	19	5		Y	Y	Y	Y	N	N	Y
3	CSA	f	18	1	Maj Dep, OCD	Y	Y	Y	Y	Y	Y	N
4	CSA	f	22	2		Y	Y	Y	Y	Y	Y	N
5	CSA	f	19	3		Y	Y	Y	Y	N	Y	N
6	CSA	f	20	2	DD-NOS, Phobia	Y	Y	Y	Y	Y	Y	Y
7	CSA	f	21	2	PTSD	Y	Y	Y	Y	Y	Y	N
8	CSA	f	18	2		Y	Y	Y	N	Y	Y	N
9	CSA	f	20	-	Maj Dep	Y	Y	N	Y	Y	Y	Y
10	VA	f	21	2	Maj Dep, PTSD, GAD	Y	Y	Y	Y	N	N	Y
11	VA	m	21	2		Y	Y	Y	Y	N	N	Y
12	VA	m	18	2	Bipolar I	Y	Y	Y	Y	N	N	N
13	C	m	18	1		Y	Y	Y	Y	N	N	N
14	C	f	19	1		Y	Y	Y	Y	N	N	N
15	C	m	19	1		Y	Y	Y	Y	N	N	Y
16	C	f	20	2		Y	Y	Y	Y	Y	Y	Y
17	C	f	20	3		Y	Y	Y	Y	Y	Y	Y
18	C	m	19	1		Y	Y	Y	Y	N	N	N
19	C	m	18	2		Y	Y	Y	Y	N	N	Y
20	C	f	19	2		Y	Y	Y	Y	Y	Y	Y
21	C	f	18	3		Y	Y	Y	Y	Y	Y	N
22	C	f	18	1		Y	Y	Y	Y	Y	Y	N
23	C	f	19	1		Y	Y	Y	Y	Y	Y	Y
24	C	f	19	3		Y	Y	Y	Y	Y	Y	Y
25	C	f	18	2		Y	Y	Y	Y	Y	Y	Y
26	C	f	18	2		Y	Y	Y	Y	Y	Y	N
27	C	f	18	2		Y	Y	Y	Y	Y	Y	Y
28	C	m	22	-		Y	Y	N	Y	N	Y	N

CSA = childhood sexual abuse, VA = verbal abuse, C = control, DD-NOS = depressive disorder – not otherwise specified, CC = corpus callosum, LVFS = lateral visual field stimulation

### Methods for the Probe Evoked Potentials

Electrodes were applied at C3 and C4, and referenced to linked ear electrodes. Ten mm gold electrodes were applied lateral to and below the right eye to monitor conjugate eye movements and blinks. All impedances were below 5 K ohms and equal bilaterally to within ± 1 K ohm. The subjects were asked to sit back in a reclining chair with a rolled towel used as a neck support. The patients fixed and maintained gaze on a mark in front of them throughout each recording period and were closely watched for eye movements.

The subjects were first asked to remember and reflect on a recent ordinary work or school situation. They were asked to raise their right hand at the wrist at the start, and to lower it when they were actively remembering the situation. If they were no longer engaged in the activity, they could signal this by raising their hand again. The recording of AEP's commenced when they lowered their hands.

Evoked potentials were recorded on a computerized EEG set to produce binaural 86 dB clicks (3 per second) and to record evoked responses for 250 msec after each click. Six hundred epochs were averaged. The low-frequency EEG filter was set at 1 Hz, the high-frequency filter at 30 Hz. Epochs greater than 15.5 μV were automatically rejected as possible artifact. Between groups, no statistical significant difference between the number of epochs recorded or rejected was observed.

Following the recording, the subject was given several queries taken from the POMS scale [64] to monitor affect. Specifically, the subject was asked to measure, on a 5-point scale from none to extreme, the level of tension, anger, sadness, hopelessness, nervousness, panic, and guilt. Subsets from the POMS scale have been used as measures of subjective mood [65].

A psychiatrist then engaged the subject in an empathic psychiatric interview lasting about 15 minutes in which the subject was asked about early family life. The psychiatrist tried to affectively engage the subject, and to get him or her to share, with emotion, a painful childhood memory. When the psychiatrist felt that the subject was affectively reexperiencing the memory, he asked the subject to try to continue to maintain his memory and mood, but without speech or motion, so that his or her evoked potentials could be measured. Following the recording, the abbreviated POMS scale was again used to measure emotional state. The unpleasant memory task was always presented after the neutral memory task because of concern that the lingering effects of the unpleasant memories would interfere with the neutral task. Following completion of the study, the psychiatrist worked with each subject to restore typical mood, and no subject left the laboratory in distress.

The averaged AEP response from each condition was printed, and all recordings were blindly read to obtain N1 and P2 peaks. N1 was defined as the maximal negative deflection between 70 and 130 msec, which conformed to expected patterns, while P2 was defined as the peak of the following positive wave. An N1-P2 measurement was made for EEG leads C3 (left side) and C4 (right side). A laterality index (LI) was calculated from these values using the formula (C3-C4)/(C3+C4) for the neutral memory and for the unpleasant memory, and these two values were subtracted from each other to give the LI from the neutral memory minus the LI from the upsetting memory (LI_AEP). When the right hemisphere was more active during the emotional than during the neutral memory, LI_AEP was < 0, and we classified responses in terms of direction of the LI_AEP (D_AEP) as either +1 for left negative HEV and -1 for right negative HEV.

### MRI imaging protocol

To measure the cerebral cortex, we used a high resolution T1-weighted MRI data set. Based on informed consent, we performed MRI only once per patient.

We scanned all patients on the same 1.5 T magnetic resonance scanner (Echospeed; General Electric Medical Systems, Milwaukee, WI, USA) equipped with a whole-body, resonant gradient set capable of echo planar imaging and a standard quadrature head coil for image detection, located at McLean Hospital, MA. MRI protocol was T1-weighted coronals (3-D, spoiled gradient recalled acquisition in the steady state [SPGR]; pulse sequence): repetition time (TR) 40 ms; echo time (TE) 5 ms; number of excitations (NEX) 2; flip angle 40°; field of view 24 cm; matrix 256 × 128; 124 slices with section thickness of 1.5 mm, no gaps. MRI data were assigned identification numbers, so that the investigators performing measurements could remain blind to any correspondence between images and subjects.

### Methods for the grey matter volume (GMV) measurements

We used 2 independent methods for determining GMV: FreeSurfer (FS) and Volumetric Brain Morphology (VBM).

The first mage analysis was performed by using a program for cortical surface-based analysis; FS which is distributed by the Massachusetts General Hospital NMR Center and CorTechs^© ^2001–2004, Boston, MA, U.S.A. [66-68]. FS is a set of semi-automated software tools used for reconstructing MR images of the cerebral cortex. All major gyrus and sulcus of the cerebral cortex were identified and traced in the flattened representation of the cerebral cortex using the program. In depth descriptions of these tools have been provided by [66-68].

The second method for measuring GMV was with VBM, which is computed using the programs SPM2 [54] and MATLAB 6.5 [69]. Briefly, images were segmented into grey matter, white matter, cerebrospinal fluid and skull/scalp compartments, then normalized to standard space and re-segmented. The spatially normalized segments of grey and white matter were smoothed using a 12-mm full-width half-maximum isotropic Gaussian kernel according to the optimized VBM protocol of Good et al [70].

### Methods for the MRI region of interest analyses

The measurements for the volumes of the hippocampus, amygdala, and corpus callosum were performed as part of another study, in submission, of 43 females, 26 with a history of childhood sexual abuse. As shown in Table 1, 19 of these subjects participated in our AEP study. Two of these subjects had corpus callosum, but not hippocampal or amygdala measurements. Hippocampus and amygdala were manually traced in their entirety according to the method detailed by Pruessner et al. [71], which used the coronal view as the default orientation but also employed saggital and horizontal views to determine specific boundaries. This technique yields excellent reliability (intra-rater ICC 0.91 to 0.95, inter-rater 0.83 to 0.94). The hippocampus was defined as including the dentate gyrus, the cornu ammonis (CA) regions, the part of the fasciolar gyrus adjacent to the CA regions, the alveus, the fimbria, and the subiculum. The posterior end of the amygdala was measured on the most posterior coronal slice where grey matter first appeared superior to the alveus (or the inferior horn of the lateral ventricle, if the alveus was not visible) and lateral to the hippocampal head. The anterior border of the amygdala was marked at the level of the closure of the lateral sulcus, and was delineated using the horizontal view. Manual tracing is currently considered optimal for measuring the volume of these two regions [72].

Anatomical measurements of corpus callosum area were obtained from the midsagittal image. An automated algorithm created in NIH Image divided the manually traced corpus callosum into seven regions as defined by Witelson [73]. Magnetic resonance image measures were performed by two independent researchers blind to all clinical variables, with interrater reliability of .83 across all regions.

### Methods for the neuropsychiatric assessments

Psychometric evaluation included the Structured Clinical Interview for DSM-IV for the diagnosis of MDD, PTSD, and other psychiatric disorders. In addition, we performed memory assessment scale (MAS) [74]. The MAS consists of 12 subtests based on the following 7 memory tasks: verbal span, list learning, prose memory, visual span, visual recognition, visual reproduction, and names-faces. The resulting global memory and summary scale scores provide measures of overall memory performance, short-term memory, verbal memory, and visual memory.

The Adult Suicidal Ideation Questionnaire (ASIQ) [75] is a 25-item self-report scale of suicidal thinking. With items rated on a 7-point Likert scale, internal consistency and test-retest reliability coefficients range from .96–.97 and .85–.95, respectively. Norms are based on 2,000 adults ages 18 years and older, which includes psychiatric outpatients, typical adults, and college students.

The Symptom Checklist-90 (SCL-90) [76] is a self-report scale used to evaluate a broad range of psychological problems and symptoms of psychopathology. The SCL-90 contains 90 items broken down into nine primary symptom dimensions. The Overall scale provides a measure of global psychological distress. More than 1,000 studies support the reliability, validity, and utility of the SCL-90.

Originally published in 1960, the Hamilton Depression Rating Scale (HAM-D) [77] was developed to assess the effectiveness of the first generation of antidepressants. Presently, the HAM-D is the most commonly used measure of depression. The scale has 17 items that evaluate depressed mood, vegetative, and cognitive symptoms of depression, and comorbid anxiety symptoms. The HAD-D is administered by a trained clinician using a semi-structured clinical interview and items are rated on either a 5-point or 3-point scale.

The Dissociative Experiences Scale (DES) [78] is a screening instrument for dissociation. The scale consists of 28 items that assess a variety of dissociative experiences, including typical ones. For each item, respondents are instructed to place a slash on a line, which is anchored at 0% on the left and 100% on the right, to show how often s/he has this experience. The DES has very good reliability and validity, including excellent construct validity.

The Mississippi Scale for Civilian PTSD (MISS) [79] is a revised non-combat version of the Mississippi Scale for Combat-Related PTSD [80]. The scale consists of 39 self-report items derived from the Diagnostic and Statistical Manual of Mental Disorders III-R criteria for PTSD. Each item is rated on a 5-point Likert scale. The MISS is internally consistent (Cronbach's a = .89 for the total scale) and split-half reliability suggests that the scale measures a single construct (PTSD), although some have questioned whether the scale measures just PTSD or PTSD plus depression [81].

### Methods for lateral visual field stimulation

The subjects were each randomly offered one of four pairs of taped glasses. Two pairs of glasses were made by covering safety glasses with white adhesive tape over one side and 50% of the medial aspect of the other. Each of these two pairs of lateral visual field glasses was taped so that it permitted vision to only either the left or the right lateral visual field as the subject was asked to fixate the center of his vision on the edge of the tape so that he was looking out of either the left half of the left eye or the right half of the right eye. The two other pairs of glasses were similar safety goggles, taped completely over one side, but only over the bottom one fourth of the other side. These glasses allowed for monocular vision, which has been shown to cause some hemispheric lateralization [82]. The tape on the bottom of the unoccluded lens gave the monocular glasses a more complex appearance.

After the first pair of glasses was worn for 2 minutes, one of the experimenters (CA) verbally asked the subject to rate his or her present feelings for each of eight affects from an abbreviated POMS scale [64], from none to extreme on a 5 point scale. The eight affects measured were: anxiety, tension, anger, sadness, hopelessness, panic, nervousness, and guilt. Following the POMS measurements, the first pair of glasses was removed, and the subject was allowed to rest for 2 minutes. Then the next pair was placed on the subject. The identical procedure was then followed for each of the 4 randomly presented pairs of glasses. We calculated a laterality index from the POMS scores reported when the subjects looked out of the left and right visual fields [(LVF-RVF)/(LVF+RVF)] for the experimental (LI_LVFS) and the monocular glasses [(L-R)/L+R)]. When more negative affect was reported from the LVF than the RVF, we assigned a negative right HEV and a left negative HEV when the LI_LVFS was < 0. We reported this categorization of the LI_LVFS as the direction of LVFS (D_LVFS) and +1 was for a right negative HEV and -1 for a left negative HEV.

Simply asking a subject to look out of one visual field or the other, in many subjects evokes a significant change in their psychological state. In clinical settings, one side is generally more neurotic and symptomatic than the other [42,43]. The subjects did nothing other than look out of one lateral visual field and then the other as described above.

With hemifield attention studies, the subject fixates both eyes at a central point and is asked to attend to a task in either the left or right half of his full visual field, as images are presented to both fields of both eyes. Monocular stimulation could be expected to activate the contralateral visual cortex since each retina is connected to both hemispheres with about a 3:2 preference for the contralateral hemisphere. With LVFS as used in this study, we believe the preference for the contralateral hemisphere is greater than with hemifield attention or with monocular stimulation because the image is presented primarily only to the nasal portion of one retina, the segment connected to the contralateral hemisphere. The taped safety glasses used in this study are not capable of limiting vision to only the nasal portion of the retina, but they can be expected to preferentially allow for stimulation of that section of the retina, and therefore might be expected to generate a stronger stimulation of the contralateral hemisphere than hemifield attention or monocular stimulation. Each subject was instructed to look with half of his eye, fixating on the edge of the tape, but we did not monitor the eye's position, and it is possible that some subjects might have been able to move their eyes so that the fixation point was lateral to the tape's edge. To the extent to which that occurred, the condition would have changed from LVFS to monocular vision, and this would have lessened contralateral hemispheric activation. Since we were correlating LVFS to other outcome measures such as probe AEP, GMV, and MAS that are variable and were not know at the time of LVFS testing, we believe that their correlations were not affected by any conscious or unconscious experimenter bias or features of the test environment.

Although visual input was presented preferentially to the contralateral hemisphere, the corpus callosum allows the transfer of information between the hemispheres. Nevertheless, an accumulating body of evidence, as discussed in the introduction and reviewed in detail elsewhere [42,43], suggests that unilateral sensory or motor stimulation can influence cognition and affect. These studies were predicated on the hypothesis that unilateral sensory stimulation would produce contralateral hemispheric activation that would, in turn, influence cognition or affect.

To test for the reliability of LVFS, the LVFS procedure was repeated between 9 to 12 months following the initial procedure to see how well the two trials correlated with each other. For each trial we calculated the differences between the abbreviated POMS scores from the LVF and the RVF stimulation, and then examined how well these differences correlated from each trial with each other. Only the first LVFS results were used in comparisons with anatomical and functional data.

### Statistical methods

All values are presented with standard deviations (± SD) and reflect means unless otherwise specified. Groups were compared using paired or unpaired t-tests, Univariate ANCOVA's, or repeated measures ANCOVA's. When we anticipated a result based on prior experimentation, we used a one-tailed test, otherwise, we used a two-tailed test. To test for bivariate correlations we used a Pearson's correlation when N was ≥ 25; otherwise, we used a Spearman's Rho. Linear regression analyses were also performed to ascertain the relationships among variables. A large number of comparisons were made in this study. To set a balance between type I and type II errors, requisite alpha p values was set to 0.01.

## Results

### Probe auditory evoked potentials

#### POMS measures of negative emotion during the memory conditions

We found that the intensity of negative affect, measured with the POMS scale after the unpleasant memory condition (14.3 ± 7.8) was significantly greater than that after the neutral memory condition (1.7 ± 2.2) by a 2-sided paired t-test (df = 27, t = -8.9, p < 0.001), but this difference was not related to grey matter volumes, measured MRI sub-cortical regions of interest, T2 relaxation times, or performance on memory tests. The intensity of the change in negative affect between conditions did not correlate directly with any psychological test parameter, although when added to regression models for the different psychological test parameters, it generally improved the models.

#### Probe Auditory Evoked Potentials during the Memory Conditions

With the probe AEP, a smaller N1-P2 measurement over either C3 (left auditory area) or C4 (right auditory area) suggests a greater degree of hemispheric involvement in the memory task. Thus, a negative laterality index (C3-C4/C3+C4) suggests that the left hemisphere is relatively more active than the right and a positive laterality index suggests that the right hemisphere is relatively more active. For the LI_AEP we subtracted the laterality index obtained during the upsetting memory from that obtained during the neutral memory. As described in detail on page 17, right negative HEV was associated with a LI_AEP with a negative value.

During the neutral memory, the 12 maltreatment subjects had a mean AEP laterality index (C3-C4/C3+C4) of -0.120 ± 0.233 and during the unpleasant memory, 0.045 ± 0.211. A paired 2-sided t-test comparing AEP laterality indices during the neutral and unpleasant conditions among the trauma subjects approached significance (p < 0.01), (t = 2.50, df = 11, P = 0.029). For the 16 control subjects, the mean during the neutral memory was -0.040 ± 0.218 and during the unpleasant memory was 0.028 ± 0.155. The differences between the two memory conditions for the control group were not significant (t = 1.008, df = 15, P = 0.33). These results replicated our 1995 findings [8].

Of the 28 subjects, 18 (64%) showed a right negative HEV. Ten subjects (36%), including 6 (38%) from the control group and 4 (33%) from the trauma group had a left negative HEV.

A grey matter volume (GMV) was calculated for each hemisphere of each patient using FS and VBM. There was a high correlation between the results from the two programs for the LH (N = 26, r = 0.91, p < 0.0001) and the RH (N = 26, r = 0.93, p < 0.0001).

**Figure 1:**
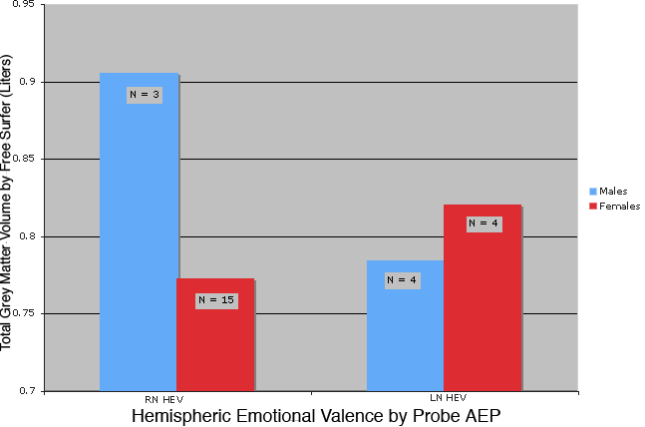
Total grey matter volume by gender and hemispheric emotional valence. This graph shows the total grey matter volume determined by FreeSurfer for male and female subjects with right and left hemispheric emotional valence by probe auditory evoked potentials.

ANCOVA models, controlling for differences in SES, showed that there were associations between GMV, HEV, and gender. As expected, there were significant gender differences with females showing an 8.4% reduction in left hemisphere GMV (F = 14.56, df = 1,21, p = 0.001) and 8.0% reduction in right hemisphere GMV (F = 10.48, df = 1,21, p = 0.004). Overall, eta-squared for effect of gender on total GMV was 0.13 indicating that it accounted for 13% of the variance in GMV. Direction of HEV was also associated with main effects that approached significance on left hemisphere GMV (F= 7.55, df = 1,21, p < 0.02), and right hemisphere GMV (F = 3.30, df = 1,21, p = 0.08). HEV direction accounted for 8% of the variance in total GMV (p < 0.05). However, there were even more marked interactions between HEV and gender. Males with right negative HEV had a 17.2% increase in left hemisphere GMV relative to males with left negative HEV, whereas right negative HEV females had a 3.3% decrease compared to females with left negative HEV (F = 15.79, df = 1,21, p = 0.001). Similarly, males with right negative HEV had a 16.3% increased in right hemisphere GMV and females with right negative HEV had a 6.1% decrease in comparison to subjects with the same gender but opposite HEV (F = 15.87, df = 1,21, p = 0.001). Overall, the interaction between gender and HEV had an eta-squared of 0.39 on total GMV. Together, gender, HEV, and gender × HEV interaction were able to account for about 60% of the individual variation in measures of gray matter volume. Figure 1 graphically shows the interaction between gender and D_AEP for total GMV from FS.

The between subjects results of a repeated measures ANOVA comparing the left and right hemispheric GMV's from FS with gender and D_AEP and their interactions as factors was highly significant for the whole model (F = 11.04, df = 3,22, p = 0.0001) as well as for the interaction between gender and D_AEP (F = 16.41, df = 1,22, p = 0.0005).

Using the GMV data from VBM, performing the same repeated measure ANOVA comparing LH and RH, we found a similar between subject interaction between gender and D_AEP (F = 23.45, df = 1,22, p = < .0001).

For the 17 females studied, controlling for intracranial volume, we found significant partial correlation coefficients (df = 14) between LI_AEP and the right (r = .67, p = .004) hippocampal. The left plus right hippocampal volumes (r = .60, p = .014) approached significance. For the left hippocampal volume, the partial correlation coefficient also approached significance (r = .47, p = .064). Since a positive LI_AEP represents a left negative HEV, these correlations indicate that as the HEV becomes more right negative, the left and right hippocampal volumes become smaller.

**Figure 2:**
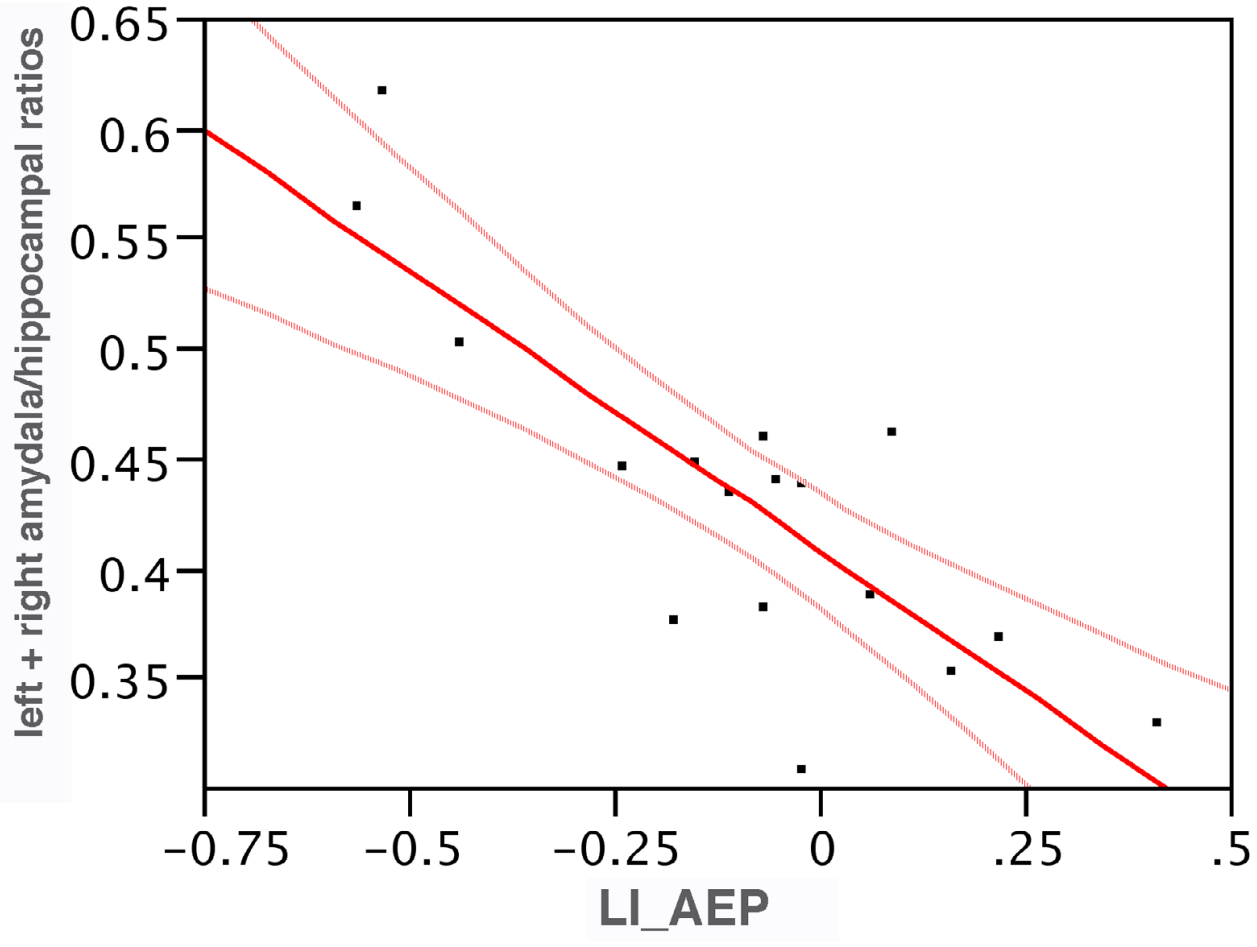
AEP by left plus right amygdala/hippocampal ratios. A scatter plot of the relation between LI_AEP and the left plus right amygdala/hippocampal ratios. (r = -.86, p = .000).

The partial correlation coefficient, again controlling for intracranial volume, between LI_AEP and the right amygdala (r = – 0.75, p = .001), and the sum of the left and right amygdala volumes (r = – .75, p = .001) were significant. This partial correlation coefficient for the left amygdala (- 0.54, p = .031) approached significance. These results indicate that as the HEV becomes more right negative, the left and right amygdala both become larger. The LI_AEP also correlated with the left amygdala volume divided by the left hippocampal volume (A/H), corrected for intracranial volumes, (r =- .69, p = .003) as well as with the right A/H ratio (r = – .87, p = .000) and with the combined left and right ratios (r = – .86, p = .000). Figure 2 show a scatter plot of the relation between LI_AEP and the left plus right A/H ratios.

We compared the T2 measurements using LIs calculated from the left and right hemispheres and observed that they were different for the left negative and right negative HEV groups. For the 17 subjects who had a right negative HEV their mean LI for T2 was 0.089 ± 0.586, and for the 10 subjects who had a left negative HEV, the mean LI was -0.366 ± 0.451, which by unpaired t-test (t = 2.11, df = 25, p = 0.045) approached significance. As shown in Table 2, the T2 relaxation times were relatively lower in the left hemisphere (LH) of the group with left negative HEV. The HEV groups did not differ significantly in the other regions of interest that we analyzed.

**Table 2:** MRI T2 relaxation times (lower values are thought to indicate greater rCBV) for the left and for right hemispheres as well as the laterality indices calculated from these values for subjects who by probe AEP had a left or a right negative hemispheric emotional valence.

Probe AEPs	N	LH	RH	LI (L-R/L+R)
Left Negative HEV	10	87.23 ± 7.51	87.56 ± 7.76	-0.366 ± 0.451
Right Negative HEV	17	89.88 ± 9.72	89.78 ± 9.54	0.089 ± 0.586*

LH = left hemisphere

RH = right hemisphere

* unpaired t-test Left – Right Negative HEV:t = 2.11, df = 25, p = 0.045.

As shown in Table 3, the visual and global memory subscales showed significant differences when the left negative HEV subjects were compared with those from the right negative HEV classification, and the verbal scale approached significance. In these 3 comparisons, the right negative HEV group performed better. None of the 4 subscales of the MAS showed differences when group (abuse or control) or gender was compared among the 28 subjects.

**Table 3:** Comparisons of the results from the Memory Assessment Scale Subscales between those subjects with Left and Right Negative HEV by Probe Auditory Evoked Potentials.

		Short-term	Verbal	Visual	Global
		
		Memory	Memory	Memory	Memory
		
Probe AEPs	N				
				
Left Negative HEV	10	109.40 ± 12.28	100.20 ± 11.02	105.40 ± 17.17	103.90 ± 15.60
Right Negative HEV	18	108.17 ± 9.41^a^	113.61 ± 13.87^b^	120.56 ± 9.33^c^	120.39 ± 10.81^d^

^a ^unpaired t-test Left – Right Negative HEV: t = -0.30, df = 26, p = 0.77, ns

^b ^unpaired t-test Left – Right Negative HEV: t = 2.62, df = 26, p = 0.014, ns

^c ^unpaired t-test Left – Right Negative HEV: t = 3.05, df = 26, p = 0.0052

^d ^unpaired t-test Left – Right Negative HEV: t = 3.30, df = 26, p = 0.0028

As reported above, correlations between LI_AEP and hippocampal volumes indicated that as the HEV becomes more right negative, the hippocampal volumes become smaller. Yet, we see here that the right negative HEV group had markedly better MAS scores. A model predicting the MAS for global memory using D_AEP (p = .008) covaried by the right hippocampus (p = 0.19) and the intracranial volumes (p = 0.65), approached significance (F (3,16) = 3.50, p = .047).

Table 4 shows the impact of considering D_AEP as an independent factor in exploring the effect of abuse history on psychiatric symptom ratings. A simple univariate ANOVA with main effects of abuse history and gender reveals only a trend level effect of abuse history on ratings of suicidaility (F(1,14) = 3.75, p = 0.073). This two factor plus interaction model also provides only a weak fit (adjusted R^2 ^= 0.051) to the available data. In contrast Univariate analysis with three main factors (abuse history, gender, D_AEP) and their interactions provides a robust fit (adjusted R^2 ^= 0.755). Taking D_AEP into consideration now reveals a marked effect of abuse on ASIQ_T scores (F(1,10) = 33.23, p < 0.001).

**Table 4:** Effect of hemispheric emotional valence on ratings of suicidaility.

Effect of including direction of hemispheric emotional valence as an independent variable when assessing the effects of abuse and gender on ratings of suicidaility.						
						
ANOVA with main effects and interactions of abuse history and gender						
						
Tests of Between-Subjects Effects						
Dependent Variable: ASIQ_T						
						
Source	Type III Sum Sq	df	Mean Square	F	Signif.	Partial h2
Corrected Model	181.167	3	60.389	1.307	0.311	0.219
GROUP	173.361	1	173.36	3.752	0.073	0.211
GENDER	2.25	1	2.25	0.049	0.829	0.003
GROUP * GENDER	4.694	1	4.694	0.102	0.755	0.007
Error	646.833	14	46.202			
Total	39470	18				
Corrected Total	828	17				
						
R Squared = .219 (Adjusted R Squared = .051)						
						
ANOVA with main effects and interactions of abuse history, gender and direction of HEV						
						
Tests of Between-Subjects Effects						
Dependent Variable: ASIQ_T						
						
Source	Type III Sum Sq	df	Mean Square	F	Signif.	Partial h2
Corrected Model	708.7	7	101.24	8.486	0.002	0.856
GROUP	396.463	1	396.46	33.23	< .001	0.769
GENDER	17.473	1	17.473	1.465	0.254	0.128
DIR_HEV	70.644	1	70.644	5.922	0.035	0.372
GROUP * GENDER	25.796	1	25.796	2.162	0.172	0.178
GROUP *DIR_HEV	56.341	1	56.341	4.723	0.055	0.321
GENDER*DIR_HEV	133.432	1	133.43	11.18	0.007	0.528
GROUP*GENDER*DIR_HEV	257.473	1	257.47	21.58	0.001	0.683
Error	119.3	10	11.93			
Total	39470	18				
Corrected Total	828	17				
						
R Squared = .856 (Adjusted R Squared = .755)						

The statistical consequences of considering D_AEP as an independent variable were most marked for ASIQ scores, but D_AEP also exerted substantial effects on many other rating scale scores. This relationship is summarized in Table 5. For each administered psychological test, the R^2 ^associated with a regression model with gender and group and their interactions as the factors was improved when D_AEP and its interactions were added to the model. The R^2 ^values by increased by 22.5% (for the HAM_D) to 1,380%, from 0.051 to 0.755 (for the ASIQ_T).

**Table 5:** This table shows summaries for five regression models each using one of the administered psychological tests as it dependent variable. Emphasis is on the effects of adding D_AEP and all its interactions to regression models after first using only gender, group and their interactions. For most dependent variables, adding D_AEP and its interactions improved the R^2 ^values as demonstrated by the significance of the F changes in the table under Model 2.

Dependent Variable	Model Summary	Model 1 Gender, Group, interactions	Model 2 Adding D_AEP and Interactions
ASIQ_T	R^2^	0.051	0.755
	F	1.310	11.060
	df	3,14	4,10
	Sig. F Change		0.001
SCL_OVR	R^2^	0.534	0.720
	F	9.180	6.020
	df	3,24	4,20
	Sig. F Change		0.002
HAM_D	R^2^	0.622	0.762
	F	13.165	2.940
	df	3,24	4,20
	Sig. F Change		0.046, ns
DES	R^2^	0.220	0.634
	F	2.263	5.647
	df	3,24	4,20
	Sig. F Change		0.003
MISS	R^2^	0.194	0.534
	F	1.930	3.640
	df	3,24	4,20
	Sig. F Change		0.022, ns

ASIQ_T = Adult suicidal ideation questionnaire, T-score

SCL_OVR = Symptoms check list for overall scale

HAM_D = Hamilton score for depression

DES = Dissociative experiences scale.

MISS = The civilian version of the Mississippi PTSD Scale

ns = not significant at the 0.01 level.

**Figure 3:**
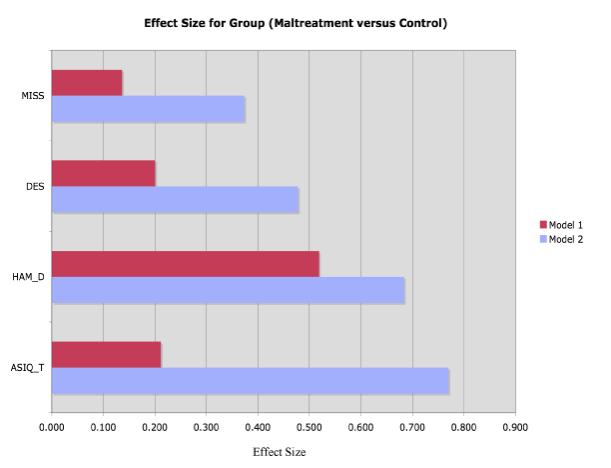
Effect size for abuse is increased when D_AEP and interactions are in model. This shows the increases in effect size for abuse when Model 1 (Univariate ANOVA with group, gender and group × gender as factors) is used compared with Model 2 (Univariate ANOVA with group, gender, D_AEP and their interactions as factors).

**Figure 4:**
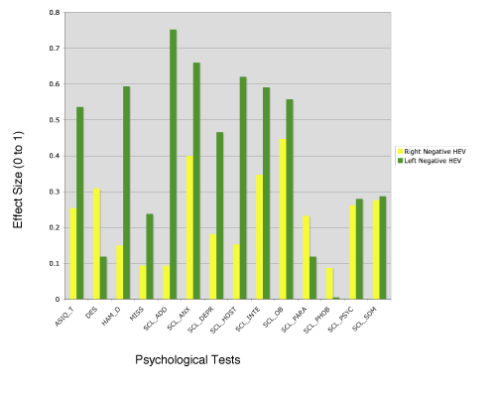
Effect size for abuse for subjects with right and for those with left negative HEV. This figure shows that the effect size for abuse (versus control) changed, sometimes drastically, when two series of univariate ANOVAs were performed with each of 14 psychological variables (ASIQ_T, DES, HAM_D, MISS, and the 10 subscales of the SCL-90) as the dependent variable and group and gender as the independent variables. In the first series (in yellow), data from the 18 subjects with a right negative HEV were used, and in the second (in green), data from the 10 subjects from the left negative HEV group were used.

Adding D_AEP not only increased R^2 ^values, but, as shown in figures 3 and 4, it also altered the effect sizes for abuse on these psychological tests.

One possible reason for the added statistical power of including D_AEP in analysis of effects of abuse history on ratings is that subjects with right negative HEV may respond differently to abuse than subjects with left negative HEV. This appears to be true, at least in a statistical sense, for many symptom scores. In general, ratings of depression (HAM-D, SCL-90 depression, SCL-90 interpersonal sensitivity, ASIQ_T), anxiety (SCL-90), attention deficit (SCL-90), hostility (SCL-90), and trauma history (MISS) were most affected by abuse history in subjects with left negative HEV. In contrast, ratings of dissociation (DES), paranoid ideation (SCL_90) and phobic anxiety (SCL90) were most affected by abuse in subjects with right negative HEV.

### Lateral visual field stimulation

A positive LI_LVFS suggests a right negative HEV because it is based on the negative emotions indicated by the POMS score when the subject looks out of the LVF (right hemisphere) minus that when he looks out the RVF. LI_AEP, on the other hand is based on the N1-P2 measurement from C3 minus that from C4, as described on page 17. A negative LI_AEP suggests a right negative HEV, and, for all 15 subjects who were given both procedures, comparing the directions of HEV between our 2 methods, AEP and LVFS, we found a highly significant correlation, (1-tailed Spearman's rho = -0.637, p = 0.005) indicating an agreement between the two methods. Overall, 9 subjects had a directional response to the glasses, in 8 of whom the direction of response concurred with direction of HEV as determined by AEP.

The HEV indicated by the control, monocular glasses did not correlate significantly with that from the AEPs (Spearman Rho = 0.13, p = 0.69). In fact, we found the control, monocular glasses to be not significant in any analyses we carried out for the LVFS glasses.

To test the reliability of LVFS, we repeated tests of LVFS, at least 9 months apart, in 14 subjects and found a Spearman's rho = .93, p = .0001 between the two tests.

**Figure 5:**
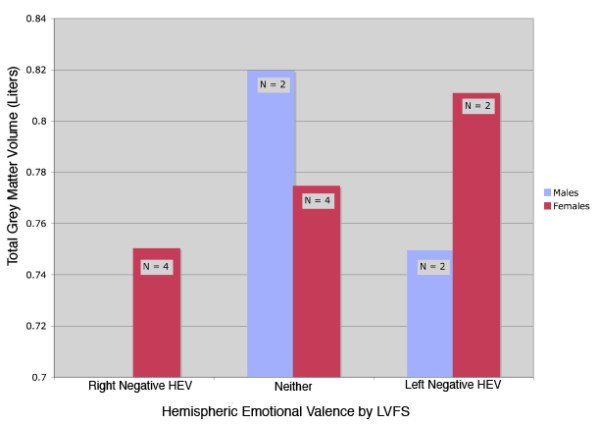
Total grey matter volume by gender and HEV by LVFS. This figure shows the total grey matter volume for males and females by the direction of their hemispheric emotional valence determined by LVFS.

There were no significant bivariate correlations between LI_LVFS and hemispheric measures of GMV by FS or VBM. As illustrated in Figure 5, for total GMV by FS, males and females have different directions of change with HEV by LVFS and the directions, though not statistically significant, are similar to those shown in Figure 1 (which illustrates GMV values categorized by the AEP procedure).

The correlation between LI_LVFS and the total white matter volume, derived from FS, approached significance (2-tailed Spearman's rho = -.54, p = .045).

The total volume for the corpus callosum correlated with the direction of LI_LVFS (n = 10, 2-tailed Spearman's Rho = -0.81, p < 0.005). The group with right negative HEV by LVFS had smaller total corpus callosi.

There were no relationships between the LVFS results and the volumes for the amygdala, the hippocampus or their ratios.

As shown in Table 6. The Spearman correlations between LI_LVFS and the left and right hemisphere T2 relaxation times and their combination approached significance. The direction of the correlations show that for both the left and right hemispheres, as with the Probe AEP procedure, those with left negative HEVs had lower T2 relaxation times than those with right negative HEVs.

**Table 6:** Show the results of 2-tailed Spearman correlations for LI_LVFS and the left and right hemispheric T2 relaxation times as well as for their sums. N = 15.

	Spearman Rho	Significance
Left Hemisphere	0.52	0.048
Right Hemisphere	0.51	0.054
Both Hemispheres	0.55	0.034

The results from the LVFS tests did not correlate with the results from the Memory Assessment Scale or the administered psychological tests.

The statistical results for LVFS came from only 15 subjects. If the probe AEP statistical tests were limited to include only the 15 subjects who received the LVFS protocol, the probe AEP would have had no statistically significant results. Having an additional 13 subjects in the AEP group allowed us to observe statistical relationships that would have been missed otherwise.

## Discussion

In the literature there has been a prevailing view that the right hemisphere is associated with the perception and expression of negative emotions [8,21,83]. Our studies, and review of the literature, suggests that this view point is overly dogmatic, and that negative valence may be left lateralized in a substantial number of individuals [44,56,57]. Further, our new data provides evidence that the degree and direction of laterality is an important individual trait, on par with gender, in the degree to which it can account for individual differences in regional brain size, functional brain activity, and psychiatric symptomatology. Although we have found gender to be an important factor in regard to HEV, we feel that a future study with larger numbers of males and females would be helpful to more precisely delineate its level of importance. In this study we did not evaluate functional imaging during the different memory conditions, but we hope to do such a study in the future.

We found that gender alone accounted for about 13% of the variance between individuals in total GMV. In contrast, direction of HEV, gender, and their interaction accounted for up to 60% of the variance. Degree and direction of HEV was also associated with substantial differences in hippocampal and amygdala volumes. Females with the most right-sided HEV tended to have the smallest hippocampal and largest amygdala volumes. (Volumes were not measured in males). Degree of HEV in AEP response accounted for 36% and 54% of the variance in hippocampal and amygdala measures, respectively.

T2 relaxation time was used as indirect measure of resting relative cerebral blood volume, with lower levels of T2 relaxation times correlating with higher blood volumes [55]. Subjects with left negative HEV had lower T2 relaxation times in their left vs. right hemisphere, suggesting greater left-sided rCBV, relative to subjects with right negative HEV.

The right negative HEV group performed significantly better on 3 of 4 memory tests. This difference persisted even when their scores were covaried for intracranial and hippocampal volumes. When direction of hemispheric emotional valence and its interactions were added to regression models examining the effects of gender and childhood maltreatment on 15 administered psychological test parameters, the R^2 ^values for 11 of the 15 models improved, and the effect sizes for the influence of childhood maltreatment were increased on most of these psychological variables.

We presented two methods for determining HEV, probe AEPs and LVFS. They provide highly correlated results, and both appear useful for delineating distinct populations of left vs. right lateralized responders. LVFS, performed in only 15 subjects (versus 28 in the AEP group), correlated with the total volume of corpus callosum, and the right and left hemispheric T2 relaxation times. The changes in GMV and T2 relaxation time by LVFS were in the same direction as those found with the AEP procedure.

Overall, having right negative HEV was associated with enhanced memory, reduced total GMV, smaller hippocampal and larger amygdala volumes (in females), and increased association between exposure to childhood abuse and symptom ratings for dissociation, paranoia and phobic avoidance. In contrast, left negative HEV was associated with diminished memory, increased total GMV, larger hippocampal and smaller amygdala volumes (in females), more left-lateralized hemispheric blood flow, and an increased association between exposure to childhood abuse and symptom ratings for suicidaility, depression, anxiety, hostility, interpersonal sensitivity, and attention deficit. We do not have an explanation for the association between enhanced memory and decreased hippocampal size, but hope that future studies clarify the effects of other factors such as lateralized blood flow and symptom ratings from abuse, and lead to further illumination.

Human biology is highly lateralized, but to varying degrees. *Situs inversus*, in which there is a mirror image reversal of thoracic and abdominal organs, affects only 0.01% of the population [84]. Between 2–6% of the population appears to have exclusively right-sided, or predominantly right-sided, language lateralization (Loring et al., 1990). Left-handedness occurs in about 12% of adults [85]. We now suggest that left-hemisphere based negative emotional valence occurs in about a third of the population and in an earlier study we reported it to occur in 40% of patients [44]. This view that negative emotional valence is lateralized, but to either right or left hemispheres, stands in marked contrast to earlier theories regarding the specialized role of the right hemispheric in the processing of negative emotions [2-5]. Assuming that negative emotional valence is exclusively right lateralized may result in research findings in which lateralized difference are either diminished or imperceptible. In contrast, recognizing that right or left-sided laterality of negative emotional valence is an important individual difference, may help to resolve discrepancies in the literature that have emerged in studies of the neurobiology and treatment of emotional disorders.

For example, there is controversy in the literature regarding the efficacy of left-sided rapid transcranial magnetic stimulation (rTMS) for treatment of refractory depression [86-88]. However we found that outcome of rTMS varies greatly depending on the whether the patient has right or left lateralized negative HEV. We predicted that stimulating a hemisphere with a positive HEV would be more likely to be helpful than stimulating one with a negative HEV. As we predicted [44], 86% percent of those with left negative HEV (based on LVFS response) had a poor outcome to a subsequent course of left prefrontal rTMS versus only 20% of subjects with right negative HEV. The right negative HEV group had a 42% mean reduction in HDRS compared to an 11% reduction in the group with left negative HEV.

We recently completed a replication of our 2002 rTMS study (unpublished observations) at MindCare Centres, British Colombia, Canada, a clinic that specializes in the treatment of depression with rTMS. Data were obtained from 23 depressed patients assessed for HEV laterality who received a 2-week course of left-sided rTMS. The right negative HEV group had a 61% decrease in their depression rating scores versus a 31% decrease in scores in the left negative HEV group. Hence, we predict that the demonstrable efficacy of rTMS could be substantially increased by selecting suitable candidates for left-sided treatment based on laterality of negative HEV, or by adjusting the side of treatment to target the hemisphere with more positive HEV.

This hypothesis may also provide a mechanistic explanation of the findings of Cohen and associates [89], who conducted a double-blind, controlled trial of high and low frequency rTMS in the treatment of 24 patients with PTSD. They found that high frequency stimulation of the right frontal region produced a more favorable outcome than high frequency stimulation of the left side. This stands in contrast to studies of depression, which favor left-sided rTMS. This difference makes sense as Schiffer [56] found that among psychotherapy patients with lateralized affective responses to LVFS, that 73% of patients with major depression (n = 15) had a right negative HEV, whereas 71% of patients with PTSD (n = 14) had a left negative HEV. Hence, left-sided rTMS should benefit most patients with depression, while right-sided rTMS should benefit most patient with PTSD. Targeting treatment to the appropriate side for each individual based on LFVS may further enhance outcomes, though this remains to be determined.

These findings may apply to other lateralized treatments such as ECT. There is evidence that psychotropic medications have lateralized effects [49,51], and several authors have predicted responses to psychotropic medications by measurement of asymmetric brain activation by dichotic listening [47,52], electroencephalogram [50,52], fMRI [53], and PET [48]. LVFS should be explored as a possible method for predicting such outcomes.

These tests for HEV were inspired by observations from split-brain studies that revealed that each hemisphere was capable of supporting independent mentation [1,38,39]. Bogen [40,41] was the first to suggest that these split-brain findings might relate to intact individuals, and his assertion is supported by a number of reports of Wada studies that found, not just affect changes, but dramatic personality changes with the anesthetization of one hemisphere [35-37,90]. For example, Masia et al reported 4 patients who recalled with severe emotional distress a major trauma such as the decapitation of a friend or an incestuous rape when one hemisphere was anesthetized, but not at baseline nor when the other hemisphere was anesthetized. The side from which the memory was released varied among patients (2 left and 2 right). Ahern et al [35] described two patients with vivid personality changes. One case went from withdrawn and sullen to affable and social following anaesthetization of the left hemisphere, and in the other went from pleasant and well adjusted to belligerent and abusive when his left hemisphere was anesthetized.

Wittling [91,92] has reported affect, blood pressure, heart rate, and cortisol changes depending on which side an upsetting film was shown to subjects. Placebo controlled studies have shown that LVFS can induce changes in affect [44,56,57] and Schiffer [42] and Morton [93] have reported not only affect but also cognitive changes with LVFS. Others [94-97] have reported similar changes following lateralized auditory stimulation in patient populations. In all of these studies, the side that induced negative affects and/or cognitions varied among individuals. Schiffer [42,43] has suggested that LVFS can be a useful adjunct to psychotherapy.

Considerable evidence indicates that unilateral sensory or motor stimulation, including LVFS, activates the contralateral hemisphere. This evidence includes studies using theta EEG [57,98], lateral ear temperature [57], BOLD fMRI [58,99], and PET [100]. The combination of lateralized psychological and physiological responses, leads Schiffer to hypothesize that LVFS might preferentially activate the contralateral hemisphere [101] and produce an associated mental state that is consistent with that hemisphere's emotional valence.

## Conclusion

Overall, the purpose of this study is to stimulate a dialogue on hemispheric emotional valence, to indicate that a substantial percent of the population may have negative emotions preferentially associated with their left hemisphere, and to suggest that this is a key individual difference with relevance to researchers in behavioral and neurosciences, as well as to clinicians treating patients with mood, anxiety and personality disorders.

## Competing interests

The author(s) declare that they have no competing interests.

## Authors' contributions

FS made substantial contributions to the conception and design of the study, the acquisition, analysis, and interpretation of data, and drafting of the manuscript. MT made substantial contributions to the conception and design of the study, analysis and interpretation of data, and revision of the manuscript. CA made substantial contributions to the acquisition of the evoked potential and LVFS data and to the acquisition, analysis, and interpretation of the functional MRI data, and to the acquisition of the anatomical MRI data. CPN made substantial contributions to the acquisition and analysis of the neuropsychological test data. AP made substantial contributions to the subject enrollments and diagnostic interviews as well as to the overall administration of the study. AT made substantial contributions to the analysis and interpretation of the grey matter volume data. SA made substantial contributions to the analysis and interpretation of the MRI region of interest data. All authors read and approved the final manuscript.
